# Prognostic significance of pretreatment elevated platelet count in patients with colorectal cancer: a meta-analysis

**DOI:** 10.18632/oncotarget.13248

**Published:** 2016-11-09

**Authors:** Yu Long, Ting Wang, Qian Gao, Chengya Zhou

**Affiliations:** ^1^ Department of Medical Oncology, Sichuan Cancer Hospital & Institute, Sichuan Cancer Center, School of Medicine, University of Electronic Science and Technology of China, Chengdu, China; ^2^ Lung Cancer Center, West China Hospital, Sichuan University, Chengdu, Sichuan, China; ^3^ Oncology Department, Du Jiang Yan Medical Center, Chengdu, Sichuan, China

**Keywords:** colorectal cancer, platelet, thrombocytosis, prognosis, meta-analysis

## Abstract

**Background:**

The prognostic effect of pretreatment elevated platelet count remains controversial in colorectal cancer patients. We conducted this meta-analysis to evaluate the prognostic impact of it in these patients.

**Methods:**

PubMed, EMBASE and Cochrane Library were searched and studies on the prognostic significance of pretreatment elevated platelet count in colorectal patients were identified. We performed the meta-analysis, using overall survival and disease-free survival as outcomes and presenting data with hazard ratio and its 95% confidence interval. Heterogeneity among studies and publication bias were also evaluated.

**Results:**

Thirty studies were included in the meta-analysis. Compared with normal platelet count, pretreatment elevated platelet count was associated with poorer overall survival (Hazard ratio = 1.837, 95% confidence interval, 1.497 to 2.255, p = 0.000) and poorer disease-free survival (Hazard ratio = 1.635, 95% confidence interval, 1.237 to 2.160, p = 0.001) in patients with colorectal cancer. In subgroup analyses, pretreatment elevated platelet count was also associated with poorer overall survival and disease-free survival in most subgroups.

**Conclusion:**

Pretreatment elevated platelet count was an independent prognostic factor of overall survival and disease-free survival in colorectal cancer patients. Large-scale prospective studies and a validation study are warranted.

## INTRODUCTION

Colorectal cancer is the third most commonly diagnosed cancer in males and the second in females, with an estimated 1.4 million cases and 693,900 deaths occurring in 2012 [1]. Several markers like carcinoembryonic antigen, C-reactive protein, albumin and tumor necrosis factors have been reported as prognostic indicators of outcomes in patients with colorectal cancer. However, it is still difficult to predict the outcome of patients before treatment.

There is a growing body of evidence showing that elevated platelet count or thrombocytosis is associated with outcomes of colorectal cancer [2–10]. Early in 1998, Monreal M et al. reported a significant association between pre-operative platelet count and survival in patients with colorectal cancer. The following studies confirmed this association [2, 3, 5, 6]. However, some other studies failed to demonstrate the association between pretreatment elevated platelet count and outcomes of colorectal cancer [11–16]. Considering the controversial evidence, we conduct a meta-analysis to evaluate whether pretreatment elevated platelet count is a prognostic marker of colorectal cancer.

## RESULTS

### Study selection

**Figure 1:**
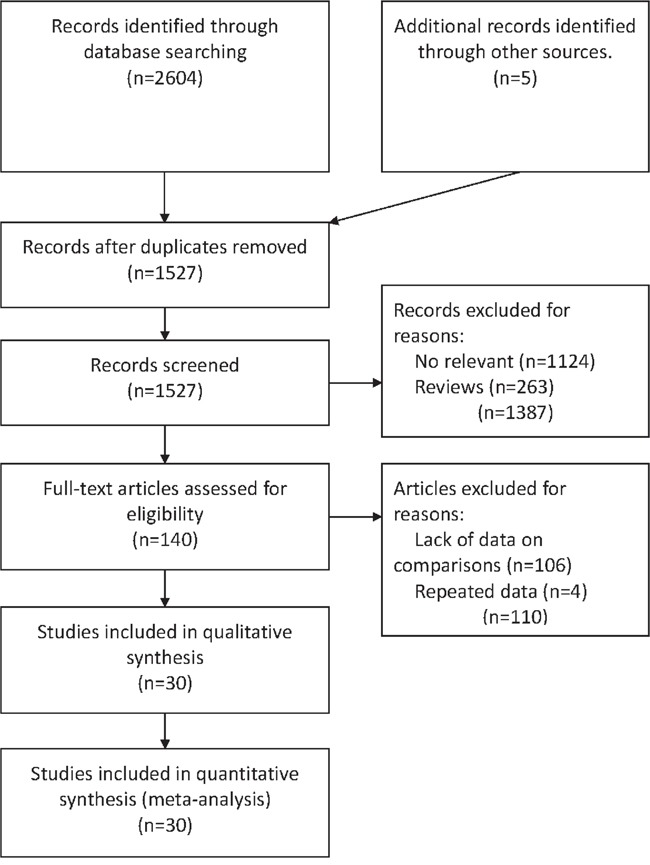
Flowchart of the process for the identification of relevant studies

Electronic search identified 2604 potentially relevant references. Additional 5 references were further identified by checking the reference list. 2469 duplicates or clearly irrelevant references were excluded through reading the abstracts. 140 references were read in full and 106 references were excluded for irrelevance or lack of data on comparisons or outcomes. Four references were excluded for repeated data. Finally, 30 references fulfilled the inclusion criteria and provided data for the meta-analysis [2–31] (Figure 1).

### Characteristics of included studies

All thirty included articles were cohort studies published from 1998 to 2016. This meta-analysis included 9123 patients. The quality score of included studies ranged from 6 to 8 stars. Hazard ratios of overall survival were available in 28 included studies and hazard ratios of disease-free survival were available in 15 included studies. Characteristics of the included studies are listed in Table 1.

**Table 1:** Characteristics of included studies

Study	Number of patients	Tumor type	Disease stage	Cut-off value	Area	Study time	Age (year)	Follow-up (year)	Treatment	Outcome	Quality score
Azab B 2014 [11]	580	colorectal cancer	I-IV	tertile	USA	2005-2011	68.6	40.5	surgery or chemotherapy	overall survival, disease-free survival	6
Baranyai Z 2014 [2]	336	colorectal cancer	I-IV	400	Hungary	2001-2011	67	36.1	surgery	overall survival, disease-free survival	8
Carruthers R 2012 [17]	115	rectal cancer	I-III	NA	UK	2000-2005	63.8	37.1	chemoradiation and surgery	overall survival, disease-free survival	6
Chen LL 2016 [3]	503	colorectal cancer	I-IV	300	China	2010-2013	58	33.8	surgery	overall survival	7
Choi KW 2014 [12]	105	colorectal cancer	I-IV	400	Korea	2005-2008	63	44	surgery	overall survival	7
Cravioto-Villanueva A 2012 [4]	163	rectal cancer	I-III	350	Mexico	2000-2007	57.6	35.4	chemoradiation and surgery	overall survival	6
Del Prete M 2015 [18]	208	colorectal cancer	IV	0.54 ULN	Italy	NA	61	NA	chemotherapy	overall survival	6
Guo T 2014 [5]	310	colorectal cancer	I-III	400	USA	2004-2013	69.9	NA	NA	overall survival	6
Jósa V 2015 [19]	336	colorectal cancer	I-IV	400	Hungary	2001-2011	66.9	NA	surgery and chemotherapy	overall survival	6
Jósa V 2015 [19]	166	colorectal cancer	IV	380	Hungary	2001-2011	62	28	surgery and chemotherapy	overall survival, disease-free survival	6
Kandemir EG 2005 [7]	198	colon cancer	I-II	400	Turkey	NA	57	47	NA	overall survival, disease-free survival	7
Kaneko M 2012 [20]	50	colorectal cancer	IV	400	Japan	2005-2010	61	17	chemotherapy	overall survival,	7
Kawai K 2013 [8]	108	rectal cancer	I-IV	365	Japan	2003-2012	63.3	22.5	chemoradiation and surgery	disease-free survival	7
Kim HJ 2015 [9]	314	rectal cancer	I-III	370	Japan	2003-2011	NA	36	chemoradiation and surgery	overall survival, disease-free survival	7
Kozak MM 2015 [21]	129	colorectal cancer	I-III	400	USA	2005-2009	67	24.7	chemotherapy	overall survival, disease-free survival	7
Kronborg CS 2015 [13]	314	colorectal cancer	IV	400	Denmark	2007-2011	64.5	21.3	chemotherapy	overall survival	7
Lee YS 2016 [22]	284	colorectal cancer	II	450	Korea	2003-2009	65	7.67	Adjuvant therapy	overall survival, disease-free survival	8
Lin MS 2012 [23]	150	colorectal cancer	I-IV	300	China	2006-2011	60.9	NA	surgery or NA	overall survival	6
Monreal M 1998 [24]	180	colorectal cancer	I-III	quertile	Spain	1994-1996	67	13	surgery and chemotherapy	overall survival	7
Neal CP 2015 [25]	302	colorectal cancer	IV	400	UK	2006-2010	64.8	29.7	surgery	overall survival	7
Paik KY 2014 [26]	600	colorectal cancer	I-IV	400	Korea	2006-2009	62.3	27.4	surgery	overall survival, disease-free survival	8
Qiu MZ 2010 [27]	363	colorectal cancer	I-IV	400	China	2005-2009	56	26	NA	overall survival	7
Roxburgh CS 2010 [28]	287	colon cancer	I-III	400	UK	1997-2005	NA	65	surgery and chemotherapy	overall survival	7
Sasaki K 2012 [29]	636	colorectal cancer	I-IV	370	Japan	2002-2008	65.9	49.1	surgery and chemotherapy	overall survival, disease-free survival	8
Shen L 2014 [14]	199	rectal cancer	II-III	300	China	2006-2011	55	31	chemoradiation and surgery	overall survival, disease-free survival	8
Song A 2015 [15]	177	colorectal cancer	IV	400	South Korea	2006-2013	52	3.1	chemotherapy	overall survival	7
Toiyama Y 2015 [30]	89	rectal cancer	I-III	300	Japan	2001-2012	65	56	chemoradiation and surgery	overall survival, disease-free survival	8
Wan S 2013 [31]	1513	colorectal cancer	I-IV	400	USA	1990-2010	64.9	54	surgery and chemotherapy	overall survival, disease-free survival	8
Wei Y 2015 [10]	286	colorectal cancer	II-III	276	China	2003-2011	62	34	surgery and chemotherapy	disease-free survival	7
Zhao H 2016 [16]	122	colorectal cancer	IV	300	China	2006-2009	NA	NA	chemotherapy	overall survival	7

NA, not available; ULN, upper limits of normal.

### Prognostic impact of pretreatment elevated platelet count on overall survival

**Figure 2:**
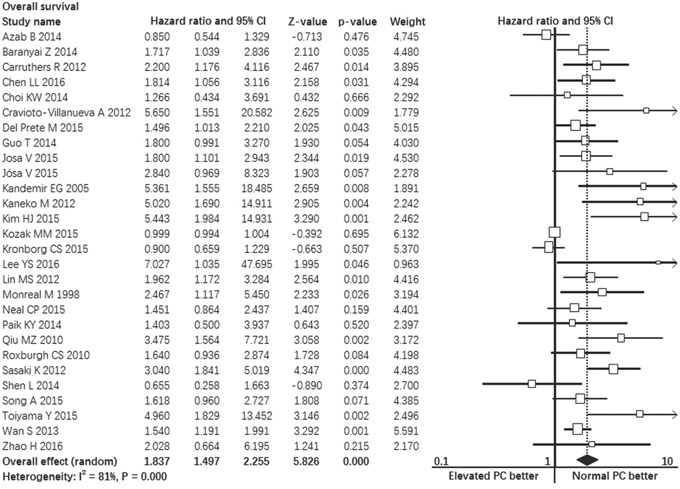
Forest plot showing the prognostic effect of pretreatment elevated platelet count on overall survival of colorectal cancer patients *CI: Confidence interval.

Twenty-eight studies contributed data to the analyses of overall survival [2-7, 9, 11-31]. Significant heterogeneity was found among studies (I^2^ = 81%, p = 0.000, Figure 2). Random-effect model was used. The pooled hazard ratio estimate showed that patients with pretreatment elevated platelet count had poorer overall survival compared with normal platelet count (HR = 1.837, 95% confidence interval, 1.497 to 2.255, p = 0.000, Figure 2).

We performed subgroup analyses on confounding factors such as disease stage, cancer type, cut-off values, etc. The results consistently showed that patients with pretreatment elevated platelet count had poorer overall survival compared with normal platelet count in most subgroups: multivariate analysis subgroup (p = 0.000), univariate analysis subgroup (p = 0.000), preoperative subgroup (p = 0.000), metastatic disease subgroup (p = 0.027), stage I-III disease subgroup (p = 0.000), rectal cancer subgroup (p = 0.009), cut-off value ≥ 400 subgroup (p = 0.000), 300 ≤ cut-off value < 400 subgroup (p = 0.000), cut-off value < 300 subgroup (p = 0.043). In colon cancer subgroup, overall survival of patients with pretreatment elevated platelet count had no difference with patients with normal platelet count (p = 0.099). The detailed results of subgroup analyses were summarized in Table 2.

**Table 2:** Summarized results of meta-analysis

Outcomes	Subgroups	Analysis Model	Studies	Heterogeneity		Hazard Ratio	95% Confidence Interval		P-value
				P-value	I-square %				
Overall survival	total	random	28	0.000	81.006	1.837	1.497	2.255	0.000
	multivariable	random	11	0.000	68.792	2.122	1.508	2.985	0.000
	univariable	fixed	8	0.162	33.341	1.675	1.331	2.108	0.000
	preoperative	random	20	0.000	83.380	2.015	1.547	2.625	0.000
	metastatic disease	random	6	0.021	62.204	1.503	1.047	2.157	0.027
	stage I-III disease	random	11	0.000	82.555	2.330	1.461	3.715	0.000
	rectal cancer	random	5	0.008	70.832	2.796	1.286	6.078	0.009
	colon cancer	random	2	0.088	65.731	2.594	0.837	8.035	0.099
	cut-off value ≥ 400 × 10^9^ / L	random	15	0.000	77.687	1.633	1.284	2.077	0.000
	300 ≤ cut-off value < 400 × 10^9^ / L	random	9	0.038	51.062	2.467	1.685	3.612	0.000
	cut-off value < 300 × 10^9^ / L	-	1	-	-	1.496	1.013	2.210	0.043
Disease-free survival	total	random	15	0.000	82.195	1.635	1.237	2.160	0.001
	multivariable	random	6	0.000	90.188	2.166	1.234	3.802	0.007
	univariable	random	9	0.008	61.329	1.377	0.992	1.909	0.056
	preoperative	random	14	0.000	83.175	1.751	1.287	2.384	0.000
	metastatic disease	-	1	-	-	0.850	0.540	1.320	0.459
	stage I-III disease	random	8	0.000	78.468	1.697	1.154	2.495	0.007
	rectal cancer	Fixed	5	0.194	34.037	1.588	1.175	2.147	0.003
	colon cancer	-	1	-	-	4.102	1.822	9.235	0.001
	cut-off value ≥ 400 × 10^9^ / L	random	6	0.000	81.695	1.589	0.989	2.555	0.056
	300 ≤ cut-off value < 400 × 10^9^ / L	Fixed	6	0.076	49.840	2.030	1.599	2.578	0.000
	cut-off value < 300 × 10^9^ / L	-	1	-	-	1.853	1.236	2.780	0.003

### Prognostic impact of pretreatment elevated platelet count on disease-free survival

**Figure 3:**
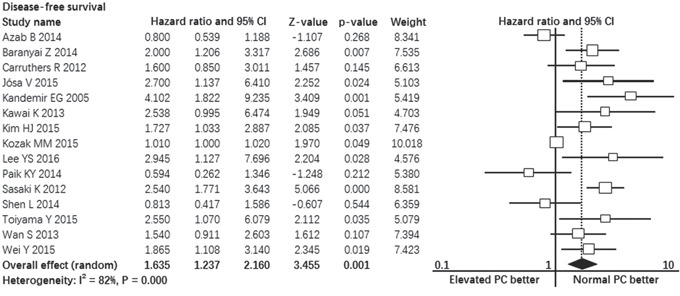
Forest plot showing the prognostic effect of pretreatment elevated platelet count on disease-free survival of colorectal cancer patients *CI: Confidence interval.

Fifteen studies contributed data to the analyses of disease-free survival. Significant heterogeneity was found among studies (I^2^ = 82%, p = 0.000, Figure 3). Random-effect model was used. The pooled hazard ratio estimate showed that patients with pretreatment elevated platelet count had poorer disease-free survival compared with normal platelet count (HR = 1.635, 95% confidence interval, 1.237 to 2.160, p = 0.001, Figure 3).

The results of subgroup analyses revealed that colorectal patients with pretreatment elevated platelet count had poorer disease-free survival compared with normal platelet count in the following subgroups: multivariate analysis subgroup (p = 0.007), preoperative subgroup (p = 0.000), stage I-III disease subgroup (p = 0.007), rectal cancer subgroup (p = 0.003), colon cancer subgroup (p = 0.001), 300 ≤ cut-off value < 400 subgroup (p = 0.000), cut-off value < 300 subgroup (p = 0.003). In three subgroups, patients with pretreatment elevated platelet count had similar disease-free survival compared with patients with normal platelet count: univariate analysis subgroup (p = 0.056), cut-off value ≥ 400 subgroup (p = 0.056) and metastatic disease subgroup (all patients received R0 resection of primary and metastasis tumors) (p = 1.320). The detailed results of subgroup analyses were summarized in Table 2.

### Publication bias

**Figure 4:**
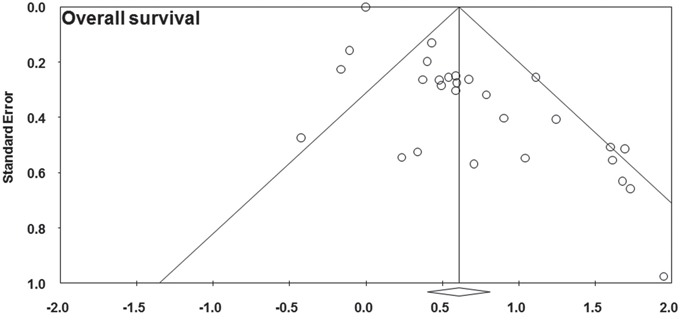
Funnel plot showing the publication bias of overall survival

**Figure 5:**
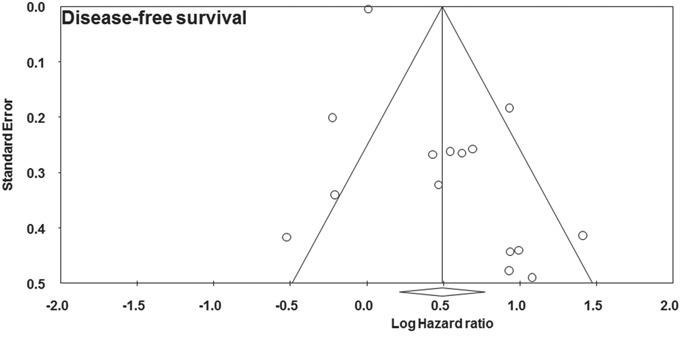
Funnel plot showing the publication bias of disease-free survival

Visual inspection of the funnel plot for overall survival and disease-free survival outcomes did not show the typically asymmetry associated with publication bias (Figure 4, Figure 5). Evidence of publication bias was also not seen with the Begg's tests of overall survival (p = 0.441) and disease-free survival (p = 1.000).

### Sensitivity analyses

**Figure 6:**
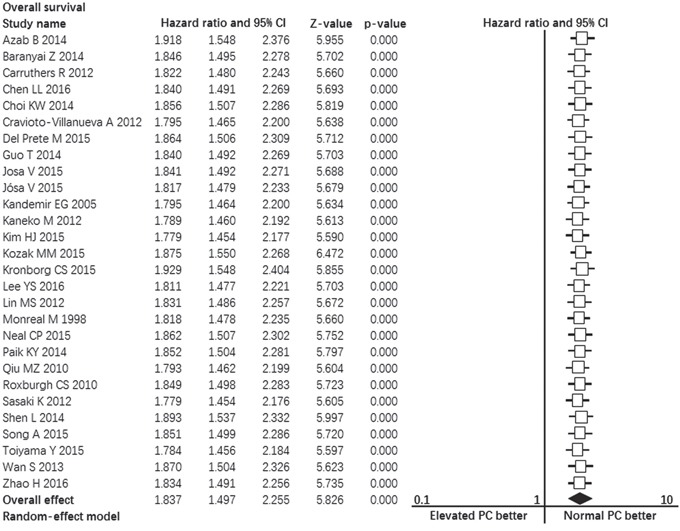
Forest plot showing the sensitivity analyses of overall survival *CI: Confidence interval

**Figure 7:**
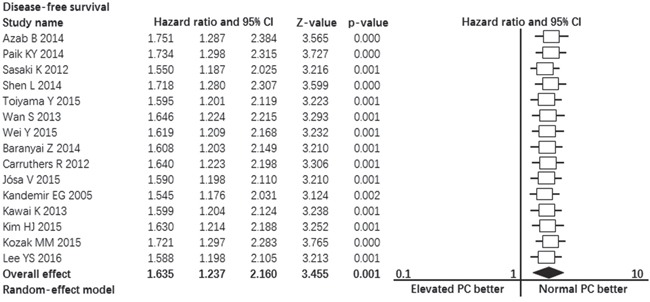
Forest plot showing the sensitivity analyses of disease-free survival *CI: Confidence interval

The result of Sensitivity analyses demonstrated that no individual study had excessive influence on the stability of the pooled effect of comparisons for overall survival (Figure 6) and disease-free survival (Figure 7). The result of this meta-analysis is robust.

## DISCUSSION

Elevated platelet count or thrombocytosis is observed in patients with various kinds of cancer and reported inversely correlated with survival [24, 32–36]. It was reported that nearly 10% to 30% of patients with colorectal cancer had elevated platelet count before treatment and worse survival than those with normal platelet count [2-5, 7, 9, 21]. In our study, the results demonstrated that elevated platelet count was associated with shorter overall survival and disease-free survival in patients with colorectal cancer. Subgroup analyses demonstrated that the impact of elevated platelet count are consistent in different disease stages, tumor locations and analysis models. The result of our study is robust. This evidence indicated that platelet could be a simple and robust prognostic marker to identify high risk patients. Those patients should be taken into account to receive adjuvant therapy or maybe anti-platelet medications. There have been accumulating evidence that postdiagnosis aspirin therapy can improve overall survival of patients with colorectal cancer in recent years [37].

It is noteworthy that the cut-off values applied in included studies were not unified. Most studies used 400 × 10^9^/L as cut-off value and some studies used 300 × 10^9^/L. The optimal cut-off value, however, was not validated in previous studies. The subgroup analyses reached consistent results among different cut-off value subgroup. The cut-off value ≥ 400 × 10^9^ / L and between 300 and 400 can both distinguished patients well by overall survival and disease-free survival. Only in cut-off value ≥ 400 × 10^9^ / L subgroup, platelet count failed to predict the disease-free survival of patients (p = 0.056, Table 2). Regarding this, it may be reasonable to suggest that the cut-off value between 300 and 400 × 10^9^ / L be applied in further investigation.

The mechanisms of tumor-related elevated platelet count or thrombocytosis remain undetermined. One of the potential hypotheses is that thrombocytosis is usually associated with inflammatory cytokines induced by interactions between tumor and host. Among these cytokines, IL-6, having multiple functions in many physiological conditions, plays a very important role in the formation of thrombocytosis [38]. By stimulating differentiation of megakaryocytes to platelets in the bone marrow, IL-6 induced thrombocytosis in various malignancies [39]. For another explanation of cancer-associated thrombocytosis, tumor can stimulate activation of platelet. As reported in several studies that cancer cells can secrete vascular endothelial growth factor to stimulates megakaryocyte differentiation [40]. For the prognostic association between elevated platelet count and patients' outcomes, there is a most widely accepted hypothesis that activated platelets contribute to the tumor growth, angiogenesis and metastasis by releasing various cytokines with inflammatory, proliferative and angiogenic activity [41–43]. With regard to tumor metastasis, platelets can cover and protect circulating tumor cells from the host's immune system. With these underlying mechanisms, platelets may be a direct or indirect target for cancer therapy.

There are some limitations of this study. First, our analysis is based on low-level evidence retrospective studies, in most of which some important confounders were not well adjusted, such as tumor stage, therapeutic strategy or ratio of colon and rectal cancer. The result of subgroup analysis, however, demonstrated that the negative prognostic significance of thrombocytosis on overall survival and disease-free survival was consistent between groups. Subgroup analysis according to therapeutic strategies was not performed because of insufficient data. Second, the sample size of some included studies were very small. The results of subgroup analyses still confirm the prognostic significance of thrombocytosis. Third, although the platelet count is easy to measure, its utility as a clinical prognostic marker could be affected by some other conditions, such as thrombosis, coronary disease, splenic disease, myeloproliferative disease, blood coagulation disorders, iron deficiency anemia and drugs. Actually, some heterogeneity was unexplainable.

In conclusion, our study demonstrated that the pretreatment elevated platelet count was an independent prognostic factor of overall survival and disease-free survival in colorectal cancer patients. It may make sense that patients with elevated platelet count should receive intensive treatment or anti-platelet therapy. And large-scale prospective studies and a validation study are warranted to confirm its prognostic significance and determine the optimal platelet cut-off value.

## MATERIALS AND METHODS

### Eligibility criteria

This meta-analysis was performed according to the statement of Preferred Reporting Items for Systematic Reviews and Meta-Analyses (PRISMA) [44]. Cohort studies, being published from inception to July 9, 2016, which reported comparisons of overall survival or disease-free survival between colorectal cancer patients with pretreatment elevated platelet count and those patients with pretreatment normal platelet count. The study participants had been pathologically diagnosed colorectal cancer patients.

### Search strategy

An electronic search in PubMed, EMBASE and the Cochrane Library were conducted from inception to July 9, 2016. The following key words in combination as medical subject heading terms and text words were used: “colorectal cancer” and “platelet” or “thrombocytosis”. Potentially relevant articles were identified by reading titles and abstracts. The full texts of the relevant articles were read to determine whether they met the inclusion criteria. The references were also searched to identify relevant studies.

### Quality assessment

For cohort studies, the 9-star Newcastle-Ottawa Scale was used to assess the risk of bias [45]. This scale is an 8-item instrument that allows for assessment of patient population and selection, study comparability, follow-up, and outcome. Interpretation of the scale is performed by awarding points for high-quality elements. Studies with 5 or more stars were defined as high-quality studies and were included.

### Statistical analyses

Data was extracted using a unified form and study information including author name, study year, study area, sample size, hazard ratio of overall survival or disease-free survival were collected. If the hazard ratio was not reported in the original article, we would calculate hazard ratio from reported data according to the methods described by Tierney et al [46]. Statistical heterogeneity among studies was examined using the Cochrane Q test by calculating the I^2^ value [47]. The I^2^ value greater than 50% or p value less than 0.05 were considered to represent significant heterogeneity. The pooled hazard ratio and the 95% confidence interval were calculated using the Z test. The pooled hazard ratio and the 95% confidence interval were calculated using the Mantel-Haenszel formula (fixed-effect model) when heterogeneity was not detected (p > 0.05), or using the DerSimonian-Laird formula (random-effect model) when heterogeneity was significant (p < 0.05) [48]. Publication bias was evaluated using the funnel plot and the Begg's test [49]. Influence analyses were conducted to access how robust the pooled estimators were by removing individual studies. An individual study was suspected of excessive influence if the point estimate of its omitted analysis was outside the 95% confidence interval of the combined analysis. Statistical analyses were performed with Comprehensive Meta Analysis professional version 2.2 (Biostat Inc, Englewood NJ, www.meta-analysis.com).

## Abbreviations

CI: Confidence interval
PRISMA: Preferred Reporting Items for Systematic Reviews and Meta-Analyses.
